# Draft genome sequence of the *Daphnia *pathogen *Octosporea bayeri*: insights into the gene content of a large microsporidian genome and a model for host-parasite interactions

**DOI:** 10.1186/gb-2009-10-10-r106

**Published:** 2009-10-06

**Authors:** Nicolas Corradi, Karen L Haag, Jean-François Pombert, Dieter Ebert, Patrick J Keeling

**Affiliations:** 1 Canadian Institute for Advanced Research, The Biodiversity Research Centre, University of British Columbia, University Boulevard, Vancouver, BC, V6T 1Z4, Canada; 2 Universität Basel, Zoologisches Institut, Evolutionsbiologie, Vesalgasse, CH-4051 Basel, Switzerland; 3 Department of Genetics, UFRGS, Porto Alegre, RS 91501-970, Brazil

## Abstract

**Background:**

The highly compacted 2.9-Mb genome of *Encephalitozoon cuniculi *placed the microsporidia in the spotlight, encoding a mere 2,000 proteins and a highly reduced suite of biochemical pathways. This extreme level of reduction is not universal across the microsporidia, with genomes known to vary up to sixfold in size, suggesting that some genomes may harbor a gene content that is not as reduced as that of *Enc. cuniculi*. In this study, we present an in-depth survey of the large genome of *Octosporea bayeri*, a pathogen of *Daphnia magna*, with an estimated genome size of 24 Mb, in order to shed light on the organization and content of a large microsporidian genome.

**Results:**

Using Illumina sequencing, 898 Mb of *O. bayeri *genome sequence was generated, resulting in 13.3 Mb of unique sequence. We annotated a total of 2,174 genes, of which 893 encodes proteins with assigned function. The gene density of the *O. bayeri *genome is very low on average, but also highly uneven, so gene-dense regions also occur. The data presented here suggest that the *O. bayeri *proteome is well represented in this analysis and is more complex that that of *Enc. cuniculi*. Functional annotation of *O. bayeri *proteins suggests that this species might be less biochemically dependent on its host for its metabolism than its more reduced relatives.

**Conclusions:**

The combination of the data presented here, together with the imminent annotated genome of *Daphnia magna*, will provide a wealth of genetic and genomic tools to study host-parasite interactions in an interesting model for pathogenesis.

## Abstract 2

The draft genome sequence of Octosporea bayeri,  a microsporidian pathogen of Daphnia, provides insights into the content and evolution of a large microsporidian genome

## Background

Microsporidia are extremely successful, highly adapted obligate intracellular parasites known to infect a wide range of animals, such as arthropods, fish, and mammals, including humans [1,2]. These parasites are characterized by the presence of a highly specialized host invasion apparatus called the polar tube (or polar filament), which is used to penetrate and infect new host cells. Microsporidian cells significantly differ from other eukaryotes, as they lack conventional mitochondria and Golgi apparatus and harbor 70S instead of 80S ribosomes [3-5]. These features were once taken to suggest that microsporidia represent a very ancient eukaryotic lineage [6-11], but recent advances in cell biology, genome sequencing, and phylogenetic reconstruction have all shown that all these apparently primitive features instead reflect an extreme state of reduction, perhaps a result of their obligate intracellular parasitic lifestyle. Instead, it is now widely acknowledged that microsporidia are, in fact, related to fungi, and have relict mitochondria (called mitosomes) [12], degenerated eukaryote-like ribosomal RNA subunits [13], and reduced genes and genomes [14-24].

The extremely reduced nature of microsporidian genomes has attracted attention since they were first noted at the end of the 1990s [13], culminating in 2001 with the completion of the first microsporidian genome from the mammalian parasite *Encephalitozoon cuniculi *[25]. The *Enc. cuniculi *genome is extremely small, at only 2.9 Mb, and the 2,000 genes it encodes provided the first compelling evidence for a strong correlation between obligate intracellular parasitism and the loss of metabolically important genes in eukaryotes. Metabolic capabilities are indeed significantly reduced in *Enc. cuniculi*, and genes required for *de novo *biosynthesis of purine and pyrimidine nucleotides or those involved in the tricarboxylic acid cycle, fatty acid beta-oxidation, respiratory electron-transport chain and the F_0_F_1_-ATPase complex are completely absent from its genome. The reduction of several metabolic pathways in *Enc. cuniculi *implied that these parasites might be extremely dependent on their host for obtaining most of their metabolites and energy. For example, it has been indeed recently demonstrated that this parasite and its mitosomes both import ATP from its host via specific transporters [26,27].

In addition to a significant reduction in its metabolic capabilities, the genome of *Enc. cuniculi *is also very compact. Its genes are reduced in size and separated by remarkably short intergenic regions. This extreme compaction has impacted the process of transcription so that in the microsporidia *Enc. cuniculi *and *Antonospora locustae *a significant part of their mRNA transcripts has been found to overlap between adjacent genes [28-30]. Genome reduction has also apparently affected the rate of gene rearrangement, as conservation of gene order is strikingly high among microsporidia compared to what has been reported for other eukaryotes [31,32].

Since the completion of the *Enc. cuniculi *genome, new genomic data from other microsporidian parasites have been limited to two in-depth genome surveys from *Enterocytozoon bieneusi *and *Nosema ceranae *[33,34], a smaller survey from *A. locustae *[32] and some very small surveys from various other species [35-38]. The deeper-sampled genomes of *Ent. bieneusi *and *A. locustae *show many similarities with that of *Enc. cuniculi *- all three genomes are compact and contain roughly the same number of genes and pathways - but this is perhaps not surprising because all three genomes are also relatively small (ranging from 2.9 to 6 Mb) and might not, therefore, represent all microsporidian genomes adequately.

So how do larger microsporidian genomes compare with smaller ones? Does their large size reflect the presence of more genes and pathways or do they harbor the same genes but separated by much larger intergenic regions? These questions have been partly addressed with genome surveys from *Spraguea lophii *[35], *Vittaforma cornea *[36], *Edhazardia aedis*, and *Brachiola algerae *[37,38], but because of their very low sequence coverage no conclusion can be drawn about their overall gene content and evolution. In the present study, we provide a 37× sequence coverage of the large genome of the microsporidian *Octosporea bayeri*. *O. bayeri *is a parasite of the freshwater planktonic crusteacean *Daphnia magna *[39]. Other *Daphnia *species have never been found to be infected. The parasite is both horizontally and vertically transmitted [40]. Vertical transmission occurs with 100% efficiency to the asexual (parthenogenetic) eggs of the host and with somewhat reduced efficiency to the sexual eggs. Horizontal transmission occurs after the host cadaver decomposes and environmental spores are released. Infection follows ingestion of spores by the filter feeding host. The parasite reduces host survival and fecundity. Its geographic distribution is limited to rock pool *D. magna *populations along the baltic Sea in Finland and Sweden [39] and a single report from the Czech Republic.

From our sequence survey, over 13 Mb of unique *O. bayeri *sequence data have been assembled and 2,174 ORFs have been identified, providing an excellent framework to characterize the overall gene content and structure of a large microsporidian genome, to compare it with its more reduced relatives and to increase the availability of genetic markers from this latter species. Consistent with small surveys from microsporidia with large genomes, the gene density of the *O. bayeri *genome is generally low but also highly variable. Most of the genes known in the *Enc. cuniculi *genome are also found in *O. bayeri*, but a number of other genes are also found that are apparently absent in other microsporidia. The functional distribution of the proteins significantly differed between *O. bayeri *and its more reduced relatives, suggesting the metabolic capacity and host dependency within the group is also variable. The wealth of genomic data from this parasite coupled with the annotation of the *Daphnia *genome should further increase the interest for this model of host-parasite interactions [41].

## Results

### Gene content of the *O. bayeri *genome

Approximately 898 Mb of DNA sequence was obtained from shotgun and paired-end 35-bp reads with the Illumina Genome Analyzer™, resulting in an estimated 34.2 to 37.2× coverage of the *O. bayeri *genome, which has been estimated to 24 Mb based on total number of bases sequenced divided by the average coverage. This calculation does not take into account the fact that some assembled contigs might represent several identical regions in the reference genome, and that unassembled reads might represent DNA sequences from other sources (that is, contaminants). Reads were assembled into 41,804 contigs representing a total of 13.3 Mb of sequence data (26% G+C), with only 20 contigs displaying evidence of contamination. The length of contigs averaged 320 bp (100 bp to a maximum of 8 kb). The small size of most contigs resulted in the incompleteness of most ORFs identified in this study and, on average, incomplete ORFs were found to encode 60% of the amino acids of their respective eukaryotic homologs. This explains why the complete (or almost complete) *O. bayeri *proteome has been identified within an assembly that is almost half the size of the estimated genome.

A total of four rRNA genes, 37 tRNAs and 2,174 predicted protein-coding ORFs were identified (Table 1). Of the *O. bayeri *ORFs, 1,405 were found to have homologs in the *Enc. cuniculi *genome, representing about 70% of its annotated genes [25] (Additional data file 1). Over 93% of *Enc. cuniculi *proteins with assigned functions and 53% of its hypothetical proteins had clear homologs in the *O. bayeri *genome [25,33]. Over 25% of *Enc. cuniculi *homologs identified are full length, while others were slightly truncated in the carboxy-terminal or amino-terminal regions, or both. Another 80 ORFs were identified that were found to have homologs in other organisms, but not *Enc. cuniculi*, 72 of which could be assigned to a functional category (Additional data file 2), the majority of which have highest similarities with fungal homologs, suggesting that they are ancestral within the lineage and not recently introduced into the *O. bayeri *genome. The remaining 689 *O. bayeri *putative ORFs (of at least 200 amino acids) returned no significant hits in BLAST homology searches against the National Center for Biotechnology Information (NCBI) non-redundant database. However, 25 of these showed significant similarities with hypothetical proteins from the *A. locustae *database, indicating that *O. bayeri *and *A. locustae *share a number of hypothetical proteins that are absent in *Enc. cuniculi *and *Ent. bieneusi*. It is also important to note that a large proportion of microsporidian hypothetical proteins have been found to be smaller than 200 amino acids [25,31-33], so the actual number of ORFs could be over 25% higher than what we report here, perhaps in the range of, or higher than, what has been recently reported for *N. ceranae *[34].

**Table 1:** General characteristics of *O. bayeri *and other microsporidian genomes

General characteristics	*O. bayeri*	*Enc. cuniculi*	*Ent. Bieneusi*
Number of chromosomes	NA	11	6
Genome size (Mb)	≤24.2*	2.9	6
Assembled Mb	13.3	2.5	3.86
Genome coverage (%)	55^†^	86	64
G+C content (%)	26	47	25
Gene density	1 per 4,593 bases^‡^	1 per 1,025 bases	1 per 1,148 bases
Mean intergenic region (bp)	429^§^	129	127
Presence of overlapping genes	No	Yes	Yes
Number of SSU-LSU rRNA genes	2^¶^	22	Unkown^¥^
Number of 5S rRNA genes	2^¶^	3	Unkown^¥^
Number of tRNAs	37	46	46
Number of tRNA synthetases	21	21	21
Number of tRNA introns (size in bp)	≥1 (50)	2 (16, 42)	2 (13, 30)
Number of splicesomal introns (size in bp)	≥6 (24-33)	13 (23-52)	19 (36-306)
Number of predicted ORFs	2,174^#^	1,997	3,804**
Number of ORFs assigned to functional categories	894 (41%)	884 (44%)	669 (39%)
Mean size of CDS (bp)	1,056^††^	1,017^††^	1,002^††^

*The genome size has been estimated using total number of bases sequenced divided by the average coverage. ^†^Based on the 24.2-Mb estimated genome size. ^‡^Based on the 200 largest contigs. ^§^Based on contigs (n = 23) in which two or more ORFs of at least 100 amino acids have been identified. ^¶^Only two contigs harboring an SSU-5.8S-LSU gene array have been identified in the *O. bayeri *genome survey. ^¥^Based on [33]. ^#^Includes ORFs with assigned functions, homologs of *Enc. cuniculi *hypothetical proteins, and hypothetical proteins of at least 200 amino acids identified in the *O. bayeri *genome. **The *Ent. bieneusi *genome has been subjected to several segmental duplications and the number of ORFs identified in that study includes a very large number of duplicates [33]. This number should, therefore, not be taken into account to determine the haploid coding capacity of this species. ^††^Based on 95 and 63 complete *Enc. cuniculi *and *Ent. bieneusi *orthologs, respectively. CDS, coding sequence.

### Functional categories represented in *O. bayeri*

**Figure 1:**
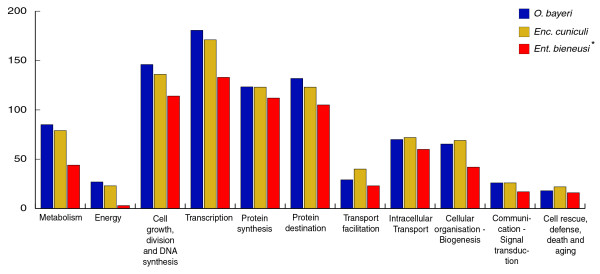
**Distribution of *O. bayeri *(blue), *Enc. cuniculi *(yellow) and *Ent. bieneusi *(red) proteins among functional categories**. The ordinate represents the number of ORFs assigned to the corresponding category. Each of the *O. bayeri *proteins was assigned to only one of eleven functional categories listed in [25,33]. The corresponding gene list is presented in the online version of this manuscript (Additional data file 3). *Based on a 4× sequence coverage [33].

**Figure 2:**
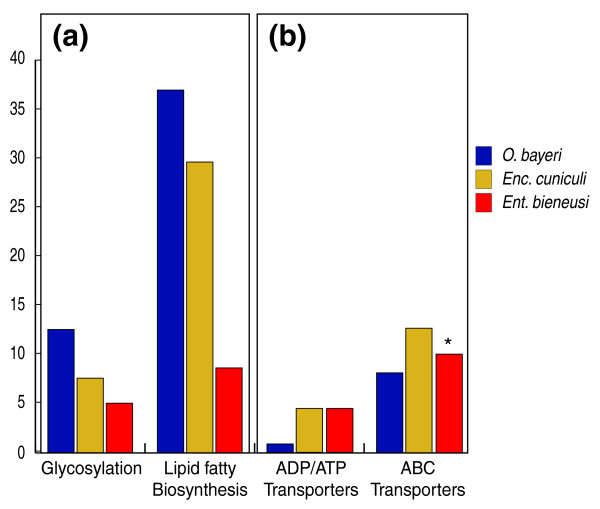
**Examples of sub-functional categories showing sharp differences in distribution between *O. bayeri *(blue), *Enc. cuniculi *(yellow) and *Ent. bieneusi *(red) proteins**. **(a) **Functional sub-categories more highly represented in *O. bayeri *than in *Enc. cuniculi *and *Ent. bieneusi*. **(b) **Functional sub-categories less represented in *O. bayeri *than in *Enc. cuniculi *and *Ent. bieneusi*. *Based on a 4× sequence coverage [33] (that is, almost 10 times lower than the present genome draft), suggesting a number of these transporters may yet be identified in the *Ent. bieneusi *genome survey.

All identified *O. bayeri *ORFs were assigned to the 11 functional categories listed in [25,33] (Figure 1; Additional data file 3). Such comparison is currently unavailable for *N. ceranae *[34]. *O. bayeri *ORFs are well distributed among the functional categories, yet display differences when compared to *Enc. cuniculi *and *Ent. bieneusi*. Specifically, five categories (metabolism, energy production, cell growth and DNA synthesis, transcription and protein destination) are more represented in *O. bayeri *than in *Enc. cuniculi *and *Ent. bieneusi*, whereas four other categories (transport facilitation, intracellular transport, cellular organization - biogenesis, and cell rescue) are reduced in number in *O. bayeri*. Within each functional category, several pathways stood out as being particularly different among the three species. For instance, genes involved in lipid and fatty acid metabolism and glycosylation were better represented in *O. bayeri *(37 and 12 proteins, respectively) than either *Enc. cuniculi *(29 and 7 proteins) or *Ent. bieneusi *(8 and 5 proteins), while proteins involved in the translocation of various substrates across membranes are underrepresented in *O. bayeri *(Figure 2). Finally, in contrast to what has been reported for other species with smaller genomes [33,34], no evidence for gene or segmental genome duplication events has been identified in the present survey.

### Phylogeny of *O. bayeri *and evolution of the ATP transporters in the microsporidia

**Figure 3:**
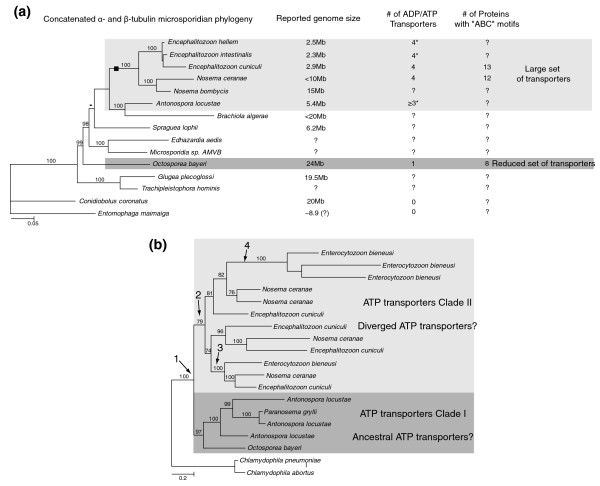
**Phylogenetic relationships of microsporidia and their ATP transporters**. **(a) **Phylogenetic reconstruction of the microsporidian phylogeny based on available α- and β-tubulin amino acid sequences and gains of ATP and ABC transporters. Known genome sizes and number of transporters are shown. *Ent. bieneusi *tubulins cluster as a sister group to the clade including *Encephalitozoon *and *Nosema *species; this position is represented by a black square. **(b) **Evolution of the ATP transporter family based on available amino acid sequences from a range of microsporidian parasites. 1, Putative ancestral duplication of ATP transporters within the microsporidia following lateral gene transfer from prokaryotes. 2, A putative secondary gene duplication occurred in the more diverged genera, *Nosema*, *Enterocytozoon *and *Encephalitozoon*. 3, Supported lineage including all three diverged genera. 4, Species-specific duplication of an ATP transporter. *Data from NC, JFP *et al*., unpublished.

*O. bayeri *was put into a phylogenetic context by comparing the amino acid sequences from its newly identified alpha- and beta-tubulins with those of other microsporidia (Figure 3a). Our tree is consistent with the most recently reported using the same amino acid sequences [42]. Specifically, *Nosema *and *Encephalitozoon *are sisters to one another, as are *Antonospora *and *Brachiola*. The remaining species all branch more deeply, and *O. bayeri *is in this tree basal to all other microsporidian species from which large genome sequence data are presently available. Only a single ATP transporter protein was identified in *O. bayeri*, and phylogenetic analyses of all presently known microsporidian members of this family show the *O. bayeri *protein clustering with strong support at the base of a clade including *Antonospora *and *Brachiola *homologues, all of which are sister to the *Encephalitozoon*/*Enterocytozoon*/*Nosema *clade (Figure 3b). This is not consistent with the rRNA tree, and might represent a mis-rooting of either tree, or ancient paralogy of the ATP transporters.

### *O. bayeri *introns

Only 13 introns have been annotated in the *Enc. cuniculi *genome at present, and we identified a total of 6 introns in the present survey, all of which are homologous to introns reported in *Enc. cuniculi *ribosomal protein genes (L19, L27a, L37a, L37, L39, S26) [25]. All the *O. bayeri *introns identified here are located within or close to the start codon, which is consistent with the introns in *Enc. cuniculi *[25], *Saccharomyces cerevisiae *[43] and cryptomonad nucleomorphs [44]. The retention of the majority of these introns leads to frame-shifts and termination codons, while their removal leads to a complete ORF that is highly conserved with homologs from other eukaryotes. The intron sequences are available with the online version of this paper (Additional data file 4).

### *O. bayeri*-specific large amino acid insertions

A number of large insertions ranging from 15 to 57 amino acids were identified in 14 conserved proteins in *O. bayeri *(O-sialoglycoprotein endopeptidase, 3-hydroxy-3-methylglutaryl CoA reductase, 3-ketoacyl CoA thiolase, α α-trehalase precursor, choline phosphate cytidyltranferase, transcription factor of the E2F/DP family, tubulin γ-chain, kinesin-like protein, pyruvate dehydrogenase E1 component subunit α, replication factor C, T complex protein 1 ε subunit, threonyl tRNA synthetase, and translation elongation factor 2). These insertions are all in-frame and in most cases are surrounded by highly conserved amino acid motifs, although they are not generally located within functionally important domains (Additional data file 5). RT-PCR confirmed that none of these inserts are removed from mRNA and so do not represent spliceosomal introns (data not shown). Similar insertions have been previously reported in the parasites *Plasmodium berghei *and *Toxoplasma gondii *[45,46].

### Length of *O. bayeri *proteins

**Figure 4:**
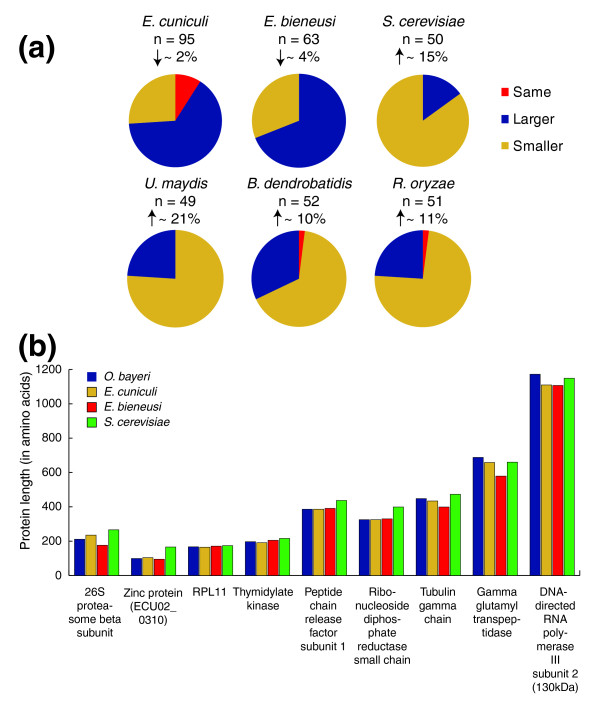
**Differences in gene length among microsporidia and their fungal relatives**. **(a) **Comparison of the length (in amino acids) of *O. bayeri *proteins to orthologs from *Enc. cuniculi*, *Ent. bieneusi*, *S. cerevisiae*, *U. maydis*, *B. dendrobatidis *and *R. oryzae*. In general, *O. bayeri *proteins are longer than microsporidian orthologues, but shorter than fungal orthologues. Vertical arrows indicate the average reduction or increase in protein size compared to *O. bayeri*. **(b) **Specific examples of length variation between orthologs from *O. bayeri*, *Enc. cuniculi*, *Ent. bieneusi *and *S. cerevisiae*.

The majority of *O. bayeri *proteins were found to be larger than homologs from *Enc. cuniculi *(69%) and *Ent. bieneusi *(65%) (Figure 4). However, the opposite trend was identified when *O. bayeri *genes were compared with other fungal lineages, in which case the majority of *O. bayeri *proteins (75% on average) were found to be smaller than homologs from the other fungal lineages, even when the fungal species compared had a smaller genome than *O. bayeri*. The difference in the number of amino acids was found to be significantly larger between *O. bayeri *and other fungal lineages (14% smaller on average) than between *O. bayeri *and other microsporidia (3% larger on average) (Figure 4).

### Gene density and synteny

**Figure 5:**
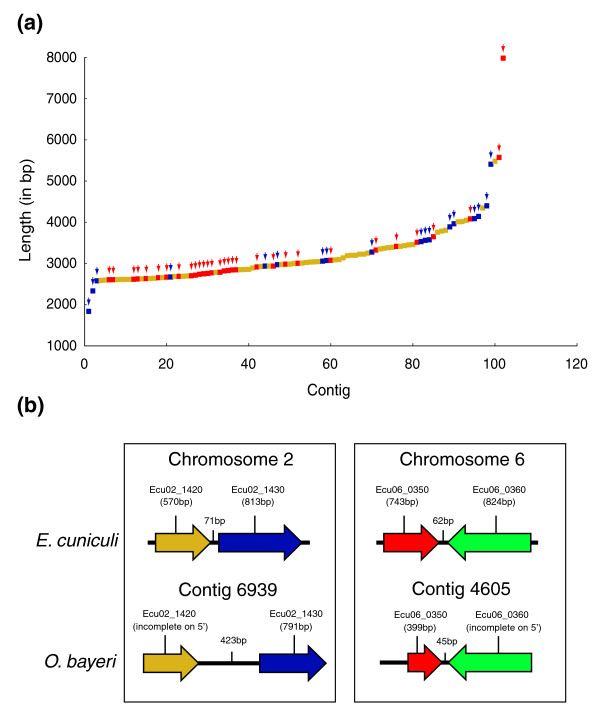
**Variation in gene density across the *O. bayeri *genome**. **(a) **Identification and distribution of ORFs (of at least 100 amino acids) among the largest *O. bayeri *contigs. Only the 100 largest contigs are shown here for convenience. Yellow dots represent contigs in which no ORF could be annotated. Blue and red arrows and dots represent contigs harboring two or one ORF, respectively. **(b) **Two cases of gene order conservation between *O. bayeri *and *Enc. cuniculi*.

Gene density and synteny in *O. bayeri *were examined by annotating all ORFs of at least 100 amino acids on the 200 largest contigs (average length of 2,795 bp). In more than half of these contigs, no putative ORF could be identified. One contig was found to harbor three putative ORFs, whereas 72 and 22 contigs harbored one or two recognizable ORFs, respectively. No correlation between the length of the contigs and the number of ORFs could be identified (Figure 5a). Based on these contigs, gene density was calculated to be 1 gene every 4,593 bases. However, when two or more ORFs were identified on the same contig the average intergenic region was calculated to be only 429 bp, suggesting the gene density is highly variable across the genome. Conservation in gene order could be identified in only two cases, representing 8% of all the gene pairs identified (Figure 5b).

### Repeated elements

The large amount of small, non-coding DNA sequences identified in this study could reflect the presence of highly repeated sequences in the *O. bayeri *genome. This possibility was investigated by measuring the sequence coverage of each contig and identifying a possible correlation with their length. As suspected, the contigs with highest coverage are also the smallest. Specifically, all contigs with a coverage over 200× are smaller than 300 bp, suggesting these are highly repetitive (Additional data file 6).

The presence of repeated elements was also investigated among all contigs. A total of 74 *O. bayeri *contigs harbor DNA segments homologous to known fungal repeated elements (Additional data file 7). The Mariner, Gypsy and Copia classes of repeated elements are the most frequently observed in *O. bayeri*. The *O. bayeri *contigs also display DNA strings that are repeated in tandem, with strings repeated at least twice identified in 1,345 contigs (data not shown). However, these tandem repeats are usually short and rarely exceed ten consecutive repeated strings. Putative stem-loop structures with AT-rich palindromic stems have been identified in a number of contigs, although the primary sequences of these potential structures, aside from their biased nucleotide composition, do not appear to be repeated *per se*.

## Discussion

### Architecture of a large microsporidian genome

The currently available microsporidian genomes best represent the lower limits in the spectrum of genome sizes, not only for Eukaryotes as a whole, but also microsporidia. The single exception to this is *N. ceranea*, whose genome is more intermediate in size, but our knowledge of microsporidian genomes is still strongly biased, which might hinder the elucidation of the evolution of this poorly understood group. Our present survey of the *O. bayeri *genome is the first deep survey of a larger microsporidian genome, and estimates from sequence coverage suggest it may even be the largest known microsporidian genome (at 24 MB). What accounts for this variation in genome size and which features of microsporidian genomes have to be reconsidered after adding a genome from the other end of the genome size spectrum? There are several answers to these questions.

Genomes might be larger due to the presence of more genes, which could be due to whole or partial genome duplications, repetitive sequences, expansion of gene families, or the retention of a greater diversity of genes in general. They might also have about the same complement of genes but have larger intergenic regions, more or larger introns, more transposable elements, and so on. Previous small-scale surveys of microsporidia with larger genomes have demonstrated a higher proportion of non-coding DNA, but reveal nothing about the overall organization of the genome because the fragments sampled were small and only a tiny fraction of the genome was characterized in any one case [35,36,38]. The data presented here provide additional evidence that large microsporidian genomes have a very low gene density, in this case up to a fivefold decrease compared to species with smaller genomes, but also provide information on the organization and structure of a large genome in this group. [25,32-34]. First, gene density is not homogeneous across the genome, but is instead a sum of long stretches (≥5.5 kb) of non-coding sequences, as well as regions where genes are separated by only 45 bp, which is even shorter than most intergenic regions found in *Enc. cuniculi*, *Ent. bieneusi *and *A. locustae*. Second, it now seems obvious that gene density alone accounts for most of the variation in genome size between different microsporidian species, although we did find numerous genes in *O. bayeri *that are absent in *Enc. cuniculi *(see below).

Smaller microsporidian genomes have also been noted as sharing a high conservation of gene order across distantly related species, which has been attributed to compaction [31,32,34]. Despite the overall low gene density, we found 8% of all annotated gene pairs (equating to 2 out of 24 gene pairs) were conserved in order between *O. bayeri *and *Enc. cuniculi*. This is not very different to what is found in other microsporidia [31,32], and close to the expectation for closely related fungi [47]. It is interesting that both cases described here involve pairs of genes that are unusually close to one another (423 and 15 bp apart). This may reflect the role of compaction in conservation of gene order, but it might also be a sampling bias since closely spaced genes are more likely to be found on the same contig in our survey, which is based on contigs, rather than a complete genome.

The large size of the *O. bayeri *genome does not reflect extensive and segmental gene duplication. However, numerous non-coding and small genomic repetitions could have played a role in its expansion. The origin of these repetitive regions is difficult to assess without a better genome assembly. Because these do not encode known functional proteins, nor harbor potential ORFs, however, it is possible that these represent telomeric and sub-telomeric regions of the *O. bayeri *genome. If this is the case, genome size variation in microsporidia could also be a consequence of variation in the size of telomeres. This prediction is supported by the recent acquisition in our laboratory of genome data from other, much smaller genomes, showing that the vast majority of unassembled Illumina™ reads belong indeed to telomeric regions (NC, JFP and PJK, unpublished).

### Length of microsporidian genes and size of the protein network

Microsporidian proteins are known to be shorter in general than orthologs in other organisms, a characteristic that has been attributed to the reduction in gene content and, by extension, protein networks in these cells [25,48]. In keeping with this, the majority of the *O. bayeri *proteins are shorter than orthologs from *S. cerevisiae *(approximately 5,570 genes, size 12 Mb), *Ustilago maydis *(approximately 6,500 genes, 20 Mb genome), *Batrachytridium dendrobatidis *(approximately 8,700 genes, 24 Mb genome) and *Rhizopus oryzae *(approximately 17,459 genes, 35 Mb genome). Interestingly, however, *O. bayeri *proteins are also larger than orthologues found in *Enc. cuniculi *and *Ent. bieneusi*. Consequently, the *O. bayeri *genome provides additional evidence that microsporidian proteins are shorter than their homologs from other fungal phyla, but also that their size correlates better with the coding capacity rather than the size of the genome in which they are found.

### Evidence for the progressive loss of ancestral genes throughout the evolution of the microsporidian lineage

Prior to this study, the vast majority of genes with predicted functions found in diverse microsporidia were also found in *Enc. cuniculi *[32-36,38]. Three exceptions were found in *A. locustae *[49-51] and a single one was found in *Ent. bieneusi *[33]. This suggested that all members of this group share a common core set of genes that have been retained after massive gene losses occurred in their ancestor, resulting in only a small degree of variability in gene content. This prediction was based, however, on a very low coverage for two large microsporidian genomes [38]. The *O. bayeri *genome and its evolutionary position within the group suggest that perhaps early microsporidians possessed many more genes with predicted functions than previously thought. It now seems likely that there was a large reduction in the ancestral proteome following the origin of microsporidia, but this was also followed by lineage-specific reductions and expansions in some branches of the microsporidian tree. The total number of ORFs identified in *O. bayeri *also suggests an overall coding capacity that is at least 10% larger than that of *Enc. cuniculi*. This is a conservative estimate based on the annotation of *O. bayeri *hypothetical proteins of at least 200 amino acids. Since it is known that *Enc. cuniculi *proteins shorter than 200 amino acids make up over a quarter of its total coding capacity [25], the overall coding capacity of *O. bayeri *is almost certainly greater still. It has been suggested that both *N. ceranae *and *Enc. bieneusi *genomes contain genes that are absent in *Enc. cuniculi*; however, the novel sequences in these genomes are apparently all hypothetical ORFs or transposable-like elements, and not genes with predicted functions. In these cases we cannot rule out that these are rapidly evolving genes with unrecognized homologues in other microsporidian genomes, or in some cases are not functional genes at all. In contrast, the genome of *O. bayeri *contains at least 80 genes with predicted functions and recognizable homologues in other organisms, but which are absent in *Enc. cuniculi*. This confirms that the proteome complexity of the ancestral microsporidian was greater than that seen in *Enc. cuniculi *(and other current taxa for which genome level surveys have been conducted to date), and suggests that further genome sequencing, especially of putatively deep-branching taxa, should reveal still more genes previously unseen in microsporidia. It is also formally possible that many genes were acquired relatively recently in the lineage leading to *O. bayeri *by lateral gene transfer, which has indeed been observed in other microsporidia [49]. However, this does not seem likely for all these genes given the rarity of transferred genes in other microsporidia, and especially given that the most highly conserved cases are all notably similar to homologs in fungi, suggesting they are more likely ancestral to the microsporidia. This implies that much more proteome diversity awaits discovery as more microsporidian genomes are characterized.

### Functional importance of *O. bayeri *proteins absent in other microsporidia

Perhaps the most intriguing finding of the present study is the identification of 80 *O. bayeri *proteins sharing homology with eukaryotes but not with *Enc. cuniculi*. Not surprisingly, these include eight transposable elements, some of which showed a high similarity to those reported from *Nosema bombycis *[52]. Transposable elements are absent in the most reduced microsporidian genomes [25,33], but are commonly reported in the ones that are larger and less compact [34,37,38,52], so in this case our study simply corroborates previous findings.

The remainder of these eukaryotic proteins stood out for being involved in important functional processes. In total, 14 are involved in transcriptional processes, including RNA polymerases or proteins involved in the transcription of tRNAs, while 19 are part of different metabolic pathways such as the metabolism of fatty acids and lipids and nucleotide metabolism. A whole set of proteins involved in the modification of proteins and three cation transporters are also present in *O. bayeri *but absent in *Enc. cuniculi*. The identification of these eukaryotic proteins is important as it shows that the *O. bayeri *proteome is more complex than that of *Enc. cuniculi *or *Ent. bieneusi*. Moreover, most of these proteins have highest similarities with homolgs from fungal lineages, suggesting they arose through common descent rather than by their recent incorporation into the genome by lateral gene transfer.

### Do *O. bayeri *protein categories reflect a lesser host dependency?

Aside from the set of *O. bayeri *proteins that are absent in *Enc. cuniculi*, the overall number of proteins with assigned functions is generally similar in the two genomes. This does not imply that both species encode the same set of identifiable eukaryotic homologs and, indeed, we observed several differences in the functional distribution of their proteins. For instance, genes involved in lipid and fatty acid metabolism are at least 25% more common in *O. bayeri *than in *Enc. cuniculi *or *Ent. bieneusi*. Similarly, *O. bayeri *harbors two additional genes involved in energy production compared to *Enc. cuniculi*, a trehalose synthase and an alternative oxidase.*O. bayeri *also harbors almost twice the number of proteins involved in glycosylation compared to *Enc. cuniculi*, suggesting a greater capacity to modify proteins, and perhaps the presence of a less simplified endoplasmic reticulum and Golgi apparatus compared to other microsporidia [3].

The presence of a larger number of genes for metabolic and energy generating proteins in *O. bayeri *does not by itself necessarily mean that this species is less dependent on its host for energy than are other microsporidia; however, we also observed a marked underrepresentation of proteins involved in stealing metabolites from the host. At the extreme, only one-quarter of the ATP transporters present in other microsporidia and around half of the *Enc. cuniculi *homologs of amino acid and sugar transporters were found in *O. bayeri*. *Octosporea *also appears to harbor a reduced set of ABC transporters compared to both *Enc. cuniculi *and *Ent. bieneusi*. Taken together, this implies that *O. bayeri *has a broader metabolic repertoire than other microsporidia while at the same time a reduced capability to derive metabolic products and energy from its host, both of which suggest it is less host-dependent than other microsporidia with smaller genomes.

The phylogenetic placement of *O. bayeri *is also consistent with the idea that host dependency evolved hand in hand with reduction in genome size and hyper-adaptation for intracellular parasitism. Indeed, *O. bayeri *clusters at a basal position in the microsporidian phylogeny, in the proximity of other species characterized by large genomes, and the only ATP transporter identified from this species was also found to be a basal representative of the gene family. If both phylogenies depict the correct evolutionary relationships within the microsporidia, then the ancestral genome of microsporidia was almost certainly large, complex, and encoded few transporters. Certainly, genome surveys of other basal representatives of the group such as *Glugea plecoglossi *or *Trachipleistophora hominis *would provide decisive evidence in support or against the evolution of reduced microsporidian genomes from larger and complex relatives. This certainly warrants further need for investigating the genomics of these highly adapted and successful parasites.

## Conclusions

Not all microsporida are characterized by small and highly reduced genomes. Here we demonstrate that the proteome complexity can vary greatly across the different species of the group, and that a larger genome size could be a good predictor of increased genomic complexity and reduced host dependency in microsporidia.

Since a microsporidian genome has now been surveyed with 454™ (*N. ceranae *[34]) and Illumina™ sequencing technology (this study), it might be interesting to compare the results. The 454™ *de novo *genome assembly of *N. ceranae *[34] resulted in lower overall sequence coverage, but an assembly of larger contigs, on average, due to the longer sequence reads. However, the Illumina™ methodology used to survey *O. bayeri *required substantially less high molecular weight DNA - in our case only 100 ng of sheared DNA. The downside of very short reads (35 bp) was mostly offset by the deep sequence coverage, allowing a detailed analysis of the coding capacity of the *O. bayeri *genome, but not of its structure (for example, conservation of gene order). Moreover, the small quantity of DNA required opens the door to genomic analyses from a broad range of uncultivatable organisms from which only a handful of contaminant-free DNA can be extracted.

Finally, an important goal of the present study was to gather a large amount of genome sequence information from *O. bayeri *so that it may complement the soon-to-be annotated genome of its exclusive host, the crustacean *D. magna*. These two species represent an excellent and well-recognized model to study host-parasite interactions [41]. The complementary nature of both genomic datasets will therefore form a great study system and provide a unique opportunity to further expand this specific field of evolutionary ecology into the post-genomic era

## Materials and methods

### DNA and RNA extraction and DNA sequencing

Total RNA and genomic DNA from *O. bayeri *(isolate OER 3-3 from the Island Oeren in the Tvärminne archipelago, southwestern Finland) were obtained from purified spores isolated from a laboratory culture of infected *D. magna *hosts (University of Basel, Switzerland). A total of 100 ng of genomic DNA was sequenced with single and paired-end 35-bp reads on the Illumina™ Genome Analyzer from Solexa (San Diego, CA, USA) by FASTERIS SA (Geneva, Switzerland). Reads were assembled using EDENA version 2.1.1, Velvet version 0.6.03 and ELAND version GAPipeline-1.0rc4 programs. This whole genome shotgun project has been deposited at GenBank under project accession [GenBank:ACSZ00000000]. The version described in this paper is the first version [Genbank:ACSZ01000000].

### Identification of *O. bayeri *homologs present in the *Enc. cuniculi *genome

The *O. bayeri *homologs that are present in the *Enc. cuniculi *genome were identified by BLAST homology searches [53] against the complete *Enc. cuniculi *genome using the NCBI BLASTALL suite. First, TBLASTX searches were performed under a cutoff *E*-value (*E *≤ 1E-10) against our local *Enc. cuniculi *database, then the *Enc. cuniculi *genes that were not found in *O. bayeri *were searched for using TBLASTX against the *O. bayeri *contigs. The *O. bayeri *tRNAs and tRNA introns identified using tRNAscan-SE and default parameters [54] were searched for in the *Enc. cuniculi *genome manually.

### Identification of *O. bayeri *eukaryotic homologs that are absent in *Enc. cuniculi*

The contigs sharing no similarities in TBLASTX searches (*E *> 1E-3) with the *Enc. cuniculi *genome have been annotated for potential ORFs using the program GETORF from the EMBOSS package [55]. Eukaryotic homologs were identified by BLASTP searches (*E *≤ 1E-10) against a local copy of the NCBI non-redundant database using the NCBI BLASTALL suite. Following the BLASTP procedure, TBLASTX searches on contigs harboring ORFs that retrieved significant BLASTP hits were performed for further validation. The resulting ORFs were assigned to functional categories using the Kyoto Encyclopedia of Genes and Genomes (KEGG) [56], Pfam [57], and UniProt [58] databases (Additional data file 2).

### Identification of putative *O. bayeri*-specific hypothetical proteins

ORFs of at least 200 amino acids that did not retrieve significant homology in BLAST searches against the *Enc. cuniculi *genome or the NCBI non-redundant database were queried against the genome survey of *A. locustae *[59] using TBLASTP searches (*E *≤ 1E-10) to identify potential hypothetical proteins of microsporidian origin. Potential functions for these ORFs were also searched for using the KEGG [56], Pfam [57], and UniProt [58] databases. ORFs of at least 200 amino acids that showed no homology in any of these searches were considered *O. bayeri*-specific putative proteins.

### Phylogenetic reconstruction

A total of 13 α- and β-tubulin amino acid sequences have been identified from a range of microsporidian species and used to reconstruct their phylogenetic relationships, as they represent the most conserved and widely sampled proteins within the group and have been successfully used in the past for similar purposes [42]. *Ent. bieneusi *tubulins have been discarded from the present phylogeny because of their extreme amino acid divergence, resulting in its unsupported positioning within the tree and in an overall reduction in the statistical support for all other phylogenetic clades. Two zygomycetes have been used as outgroups as this phylum has been proposed to represent the most recent fungal common ancestor of microsporidia [20,22]. The α- and β-tubulin amino acid sequences were aligned using Muscle v3.7 [60] and the most conserved regions selected using Gblocks 0.91b [61]. The microsporidia phylogeny was reconstructed using concatenated α- and β-tubulin amino acid sequences and MrBayes v 3.1.2 [62] with six General Time Reversible (GTR) types of substitutions, Dayoff acid substitution model and invariable plus gamma rate variations across sites. The Markov chain Monte Carlo search was run for 10,000 generations, sampling the Markov chain every 10 generations, and 250 were discarded as 'burn-in'. The relationships among microsporidia ATP transporters were studied in parallel using amino acid sequences retrieved from public databases and the parameters explained above.

### Introns, gene density, and gene length

The *O. bayeri *ORFs with assigned functions were screened for potential frameshit mutations caused by the potential presence of introns, with introns previously reported in *Enc. cuniculi *[25] searched for manually. Gene density in the *O. bayeri *genome was determined by annotating ORFs of at least 100 amino acids along the 200 largest contigs used in this study. A number of complete *O. bayeri *proteins have been compared against orthologs from *Enc. cuniculi*, *Ent. bieneusi*, *S. cerevisiae*, *Neurospora crassa*, *U. maydis*, *B. dendrobatidis *and *R. oryzae *to identify the presence of significant differences in gene length. *O. bayeri*-specific inserts in otherwise highly conserved proteins were screened for by visual inspection of BLAST search results, compared with orthologs using MEGA 4 [63], and their presence in mRNAs confirmed by RT-PCR. Locations of the *O. bayeri*-specific inserts on the corresponding protein three-dimensional structures were determined using SwissPDB-viewer and QuickPDB from the RSCB Protein Data Bank for available structures.

### Repeated elements

DNA regions in the *O. bayeri *contigs showing homology with fungal repeated elements were identified with CENSOR [64] from the Genetic Information Research Institute webserver. Repeated elements arrayed in tandem in the *O. bayeri *contigs were determined with Tandem Repeat Finder 4.03 [65] using a match/mismatch/indel ratio of 2/7/7 and a minimum score of 50. Putative stem-loop structures in the *O. bayeri *contigs were screened for with PALINDROME from the EMBOSS package using a minimum stem length of 10 and a maximum loop length of 4.

## Abbreviations

GTR: General Time Reversible; KEGG: Kyoto Encyclopedia of Genes and Genomes; NCBI: National Center for Biotechnology Information; ORF: open reading frame.

## Authors' contributions

NC conceived the study, performed molecular and bioinformatics analyses, contributed major scientific ideas and drafted the manuscript. KLH cultured *O. bayeri *strains and provided DNA and RNA samples required for sequencing. JFP performed bioinformatics analyses and drafted the manuscript. DE provided the raw sequence data on which all presented analyses have been performed and drafted the manuscript. PJK contributed to scientific ideas presented here and in conceiving the study, and drafted the manuscript.

## Additional data files

The following additional data are available with the online version of this paper: a table listing *Enc. cuniculi *predicted genes and the putative counterparts we identified in *O. bayeri *(Additional data file 1); a table listing the 80 *O. bayeri *proteins with assigned functions and motifs that are absent in *Enc. cuniculi *(Additional data file 2); a table listing *O. bayeri *ORFs and their assignment to functional categories (according to [25]) (Additional data file 3); the sequences of the six introns identified in *O. bayeri *(Additional data file 4); a figure showing three examples of large gene inserts we identified in otherwise conserved eukaryotic proteins (Additional data file 5); a graphical representation of the number of contigs used in this study and their respective sequence coverage (Additional data file 6); list of a number of repetitive elements we identified in the *O. bayeri *genome (Additional data file 7).

## Supplementary Material

Additional data file 1 *Enc. cuniculi *predicted genes and the putative counterparts identified in *O. bayeri*. Click here for file

Additional data file 2 The 80 *O. bayeri *proteins with assigned functions and motifs that are absent in *Enc. cuniculi*. Click here for file

Additional data file 3 *O. bayeri *ORFs and their assignment to functional categories (according to [25]). Click here for file

Additional data file 4 Sequences of the six introns identified in *O. bayeri*. Click here for file

Additional data file 5 Three examples of large gene inserts we identified in otherwise conserved eukaryotic proteins. Click here for file

Additional data file 6 The number of contigs used in this study and their respective sequence coverage. Click here for file

Additional data file 7 Repetitive elements we identified in the *O. bayeri *genome. Click here for file

## References

[B1] Becnel JJ Andreadis TG Witter M, Weiss LM Microsporidia in insects. The Microsporidia and Microsporidiosis 1999 Washington, DC: American Society of Microbiology Press 447 501

[B2] Larsson JIR Identification of microsporidia. Acta Protozoologica 1999 38 161 197

[B3] Vávra J Larsson JIR Wittner M, Weiss LM Structure of the microsporidia. The Microsporidia and Microsporidiosis 1999 Washington, DC: ASM Press 7 84

[B4] Ishihara R Hayashi YJ Some properties of ribosomes from the sporoplasm of *Nosema bombycis*. Invert Pathol 1968 11 377 385 10.1016/0022-2011(68)90186-9

[B5] Curgy JJ Vávra J Vivarès CP Presence of ribosomal RNAs with prokaryotic properties in Microsporidia, eukaryotic organisms. Biol Cell 1980 38 49 51

[B6] Brown JR Doolittle WF Root of the universal tree of life based on ancient aminoacyl-tRNA synthetase gene duplications. Proc Natl Acad Sci USA 1995 92 2441 2445 10.1073/pnas.92.7.2441 7708661 PMC42233

[B7] Cavalier-Smith T Eukaryote kingdoms: seven or nine? Biosystems 1981 14 461 481 10.1016/0303-2647(81)90050-2 7337818

[B8] Cavalier-Smith T Eukaryotes with no mitochondria. Nature 1987 326 332 333 10.1038/326332a0 3561476

[B9] Kamaishi T Hashimoto T Nakamura Y Masuda Y Nakamura F Okamoto K Shimizu M Hasegawa M Complete nucleotide sequences of the genes encoding translation elongation factors 1 alpha and 2 from a microsporidian parasite, *Glugea plecoglossi*: implications for the deepest branching of eukaryotes. J Biochem 1996 120 1095 1103 9010756 10.1093/oxfordjournals.jbchem.a021527

[B10] Kamaishi T Hashimoto T Nakamura Y Nakamura F Murata S Okada N Okamoto K Shimizu M Hasegawa M Protein phylogeny of translation elongation factor EF-1 alpha suggests microsporidians are extremely ancient eukaryotes. J Mol Evol 1996 42 257 263 10.1007/BF02198852 8919877

[B11] Vossbrinck CR Maddox JV Friedman S Debrunner-Vossbrinck BA Woese CR Ribosomal RNA sequence suggests microsporidia are extremely ancient eukaryotes. Nature 1987 326 411 414 10.1038/326411a0 3550472

[B12] Williams BA Hirt RP Lucocq JM Embley TM A mitochondrial remnant in the microsporidian *Trachipleistophora hominis*. Nature 2002 418 865 869 10.1038/nature00949 12192407

[B13] Biderre C Peyretaillade E Duffieux F Peyret P Metenier G Vivares C The rDNA unit of *Encephalitozoon cuniculi *(Microsporidia): complete 23S sequence and copy number. J Eukaryot Microbiol 1997 44 76S 10.1111/j.1550-7408.1997.tb05790.x 9508456

[B14] Brown JR Doolittle WF Gene descent, duplication, and horizontal transfer in the evolution of glutamyl- and glutaminyl-tRNA synthetases. J Mol Evol 1999 49 485 495 10.1007/PL00006571 10486006

[B15] Cavalier-Smith T Only six kingdoms of life. Proc Biol Sci 2004 271 1251 1262 10.1098/rspb.2004.2705 15306349 PMC1691724

[B16] Corradi N Keeling PJ Microsporidia: a journey through radical taxonomical revisions. Fungal Biol Rev 2009 doi: 10.1016/j.fbr.2009.05.001.

[B17] Edlind TD Li J Visvesvara GS Vodkin MH McLaughlin GL Katiyar SK Phylogenetic analysis of beta-tubulin sequences from amitochondrial protozoa. Mol Phylogenet Evol 1996 5 359 367 10.1006/mpev.1996.0031 8728394

[B18] Fast NM Logsdon JM Jr Doolittle WF Phylogenetic analysis of the TATA box binding protein (TBP) gene from *Nosema locustae*: evidence for a microsporidia-fungi relationship and spliceosomal intron loss. Mol Biol Evol 1999 16 1415 1419 10563021 10.1093/oxfordjournals.molbev.a026052

[B19] Hirt RP Logsdon JM Jr Healy B Dorey MW Doolittle WF Embley TM Microsporidia are related to Fungi: evidence from the largest subunit of RNA polymerase II and other proteins. Proc Natl Acad Sci USA 1999 96 580 585 10.1073/pnas.96.2.580 9892676 PMC15179

[B20] Keeling PJ Congruent evidence from alpha-tubulin and beta-tubulin gene phylogenies for a zygomycete origin of microsporidia. Fungal Genet Biol 2003 38 298 309 10.1016/S1087-1845(02)00537-6 12684019

[B21] Keeling PJ Luker MA Palmer JD Evidence from beta-tubulin phylogeny that microsporidia evolved from within the fungi. Mol Biol Evol 2000 17 23 31 10666703 10.1093/oxfordjournals.molbev.a026235

[B22] Lee SC Corradi N Byrnes EJ 3rd Torres-Martinez S Dietrich FS Keeling PJ Heitman J Microsporidia evolved from ancestral sexual fungi. Curr Biol 2008 18 1675 1679 10.1016/j.cub.2008.09.030 18976912 PMC2654606

[B23] Thomarat F Vivares CP Gouy M Phylogenetic analysis of the complete genome sequence of *Encephalitozoon cuniculi *supports the fungal origin of microsporidia and reveals a high frequency of fast-evolving genes. J Mol Evol 2004 59 780 791 10.1007/s00239-004-2673-0 15599510

[B24] Peer Y Van de Ben Ali A Meyer A Microsporidia: accumulating molecular evidence that a group of amitochondriate and suspectedly primitive eukaryotes are just curious fungi. Gene 2000 246 1 8 10.1016/S0378-1119(00)00063-9 10767522

[B25] Katinka MD Duprat S Cornillot E Metenier G Thomarat F Prensier G Barbe V Peyretaillade E Brottier P Wincker P Delbac F El Alahoui H Peyret P Saurin W Gouy M Weissenbach J Vivarés CP Genome sequence and gene compaction of the eukaryote parasite *Encephalitozoon cuniculi*. Nature 2001 414 450 453 10.1038/35106579 11719806

[B26] Tsaousis AD Kunji ER Goldberg AV Lucocq JM Hirt RP Embley TM A novel route for ATP acquisition by the remnant mitochondria of *Encephalitozoon cuniculi*. Nature 2008 453 553 556 10.1038/nature06903 18449191

[B27] Williams BA Haferkamp I Keeling PJ An ADP/ATP-specific mitochondrial carrier protein in the microsporidian *Antonospora locustae*. J Mol Biol 2008 375 1249 1257 10.1016/j.jmb.2007.11.005 18078956

[B28] Corradi N Burri L Keeling PJ mRNA processing in *Antonospora locustae *spores. Mol Genet Genomics 2008 280 565 574 10.1007/s00438-008-0387-5 18818951

[B29] Corradi N Gangaeva A Keeling PJ Comparative profiling of overlapping transcription in the compacted genomes of microsporidia *Antonospora locustae *and *Encephalitozoon cuniculi*. Genomics 2008 91 388 393 10.1016/j.ygeno.2007.12.006 18280697

[B30] Williams BA Slamovits CH Patron NJ Fast NM Keeling PJ A high frequency of overlapping gene expression in compacted eukaryotic genomes. Proc Natl Acad Sci USA 2005 102 10936 10941 10.1073/pnas.0501321102 16037215 PMC1182411

[B31] Corradi N Akiyoshi DE Morrison HG Feng X Weiss LM Tzipori S Keeling PJ Patterns of genome evolution among the microsporidian parasites *Encephalitozoon cuniculi*, *Antonospora locustae *and *Enterocytozoon bieneusi*. PLoS ONE 2007 2 e1277 10.1371/journal.pone.0001277 18060071 PMC2099475

[B32] Slamovits CH Fast NM Law JS Keeling PJ Genome compaction and stability in microsporidian intracellular parasites. Curr Biol 2004 14 891 896 10.1016/j.cub.2004.04.041 15186746

[B33] Akiyoshi DE Morrison HG Lei S Feng X Zhang Q Corradi N Mayanja H Tumwine JK Keeling PJ Weiss LM Tzipori S Genomic survey of the non-cultivatable opportunistic human pathogen, *Enterocytozoon bieneusi*. PLoS Pathog 2009 5 e1000261 10.1371/journal.ppat.1000261 19132089 PMC2607024

[B34] Cornman RS Chen YP Schatz MC Street C Zhao Y Desany B Egholm M Hutchison S Pettis JS Lipkin WI Evans JD Genomic analyses of the microsporidian *Nosema ceranae*, an emergent pathogen of honey bees. PLoS Pathog 2009 5 e1000466 10.1371/journal.ppat.1000466 19503607 PMC2685015

[B35] Hinkle G Morrison HG Sogin ML Genes coding for reverse transcriptase, DNA-directed RNA polymerase, and chitin synthase from the microsporidian *Spraguea lophii*. Biol Bull 1997 193 250 251 9390396 10.1086/BBLv193n2p250

[B36] Mittleider D Green LC Mann VH Michael SF Didier ES Brindley PJ Sequence survey of the genome of the opportunistic microsporidian pathogen, *Vittaforma corneae*. J Eukaryot Microbiol 2002 49 393 401 10.1111/j.1550-7408.2002.tb00218.x 12425527

[B37] Gill EE Becnel JJ Fast NM ESTs from the microsporidian *Edhazardia aedis*. BMC Genomics 2008 9 296 10.1186/1471-2164-9-296 18570666 PMC2474625

[B38] Williams BA Lee RC Becnel JJ Weiss LM Fast NM Keeling PJ Genome sequence surveys of *Brachiola algerae *and *Edhazardia aedis *reveal microsporidia with low gene densities. BMC Genomics 2008 9 200 10.1186/1471-2164-9-200 18445287 PMC2387174

[B39] Ebert D Ecology, Epidemiology, and Evolution of Parasitism in Daphnia [Internet] Bethesda (MD): National Library of Medicine (US), National Center for Biotechnology Information http://www.ncbi.nlm.nih.gov/entrez/query.fcgi?db=Books

[B40] Vizoso DB Lass S Ebert D Different mechanisms of transmission of the microsporidium *Octosporea bayeri*: a cocktail of solutions for the problem of parasite permanence. Parasitology 2005 130 501 509 10.1017/S0031182004006699 15991493

[B41] Ebert D Host-parasite coevolution: insights from the *Daphnia*-parasite model system. Curr Opin Microbiol 2008 11 290 301 10.1016/j.mib.2008.05.012 18556238

[B42] Lee RC Williams BA Brown AM Adamson ML Keeling PJ Fast NM Alpha- and beta-tubulin phylogenies support a close relationship between the microsporidia *Brachiola algerae *and *Antonospora locustae*. J Eukaryot Microbiol 2008 55 388 392 19017059 10.1111/j.1550-7408.2008.00348.x

[B43] Spingola M Grate L Haussler D Ares M Jr Genome-wide bioinformatic and molecular analysis of introns in *Saccharomyces cerevisiae*. RNA 1999 5 221 234 10.1017/S1355838299981682 10024174 PMC1369754

[B44] Douglas S Zauner S Fraunholz M Beaton M Penny S Deng LT Wu X Reith M Cavalier-Smith T Maier UG The highly reduced genome of an enslaved algal nucleus. Nature 2001 410 1091 1096 10.1038/35074092 11323671

[B45] Clarke JL Sodeinde O Mason PJ A unique insertion in *Plasmodium berghei *glucose-6-phosphate dehydrogenase-6-phosphogluconolactonase: evolutionary and functional studies. Mol Biochem Parasitol 2003 127 1 8 10.1016/S0166-6851(02)00298-0 12615331

[B46] Yang S Parmley SF *Toxoplasma gondii *expresses two distinct lactate dehydrogenase homologous genes during its life cycle in intermediate hosts. Gene 1997 184 1 12 10.1016/S0378-1119(96)00566-5 9016946

[B47] Seoighe C Federspiel N Jones T Hansen N Bivolarovic V Surzycki R Tamse R Komp C Huizar L Davis RW Scherer S Tait E Shaw DJ Harris D Murphy L Oliver K Taylor K Rajandream MA Barrell BG Wolfe KH Prevalence of small inversions in yeast gene order evolution. Proc Natl Acad Sci USA 2000 97 14433 14437 10.1073/pnas.240462997 11087826 PMC18936

[B48] Zhang J Protein-length distributions for the three domains of life. Trends Genet 2000 16 107 109 10.1016/S0168-9525(99)01922-8 10689349

[B49] Fast NM Law JS Williams BA Keeling PJ Bacterial catalase in the microsporidian *Nosema locustae*: implications for microsporidian metabolism and genome evolution. Eukaryot Cell 2003 2 1069 1075 10.1128/EC.2.5.1069-1075.2003 14555490 PMC219363

[B50] Slamovits CH Keeling PJ Class II photolyase in a microsporidian intracellular parasite. J Mol Biol 2004 341 713 721 10.1016/j.jmb.2004.06.032 15288781

[B51] Williams BA Keeling PJ Microsporidian mitochondrial proteins: expression in *Antonospora locustae *spores and identification of genes coding for two further proteins. J Eukaryot Microbiol 2005 52 271 276 15927004 10.1111/j.1550-7408.2005.05-00036.x

[B52] Xu J Pan G Fang L Li J Tian X Li T Zhou Z Xiang Z The varying microsporidian genome: existence of long-terminal repeat retrotransposon in domesticated silkworm parasite *Nosema bombycis*. Int J Parasitol 2006 36 1049 1056 10.1016/j.ijpara.2006.04.010 16797019

[B53] Altschul SF Gish W Miller W Myers EW Lipman DJ Basic local alignment search tool. J Mol Biol 1990 215 403 410 2231712 10.1016/S0022-2836(05)80360-2

[B54] Lowe TM Eddy SR tRNAscan-SE: a program for improved detection of transfer RNA genes in genomic sequence. Nucleic Acids Res 1997 25 955 964 10.1093/nar/25.5.955 9023104 PMC146525

[B55] Rice P Longden I Bleasby A EMBOSS: the European Molecular Biology Open Software Suite. Trends Genet 2000 16 276 277 10.1016/S0168-9525(00)02024-2 10827456

[B56] Ogata H Goto S Sato K Fujibuchi W Bono H Kanehisa M KEGG: Kyoto Encyclopedia of Genes and Genomes. Nucleic Acids Res 1999 27 29 34 10.1093/nar/27.1.29 9847135 PMC148090

[B57] Finn RD Tate J Mistry J Coggill PC Sammut SJ Hotz HR Ceric G Forslund K Eddy SR Sonnhammer EL Bateman A The Pfam protein families database. Nucleic Acids Res 2008 36 D281 288 10.1093/nar/gkm960 18039703 PMC2238907

[B58] Apweiler R Bairoch A Wu CH Barker WC Boeckmann B Ferro S Gasteiger E Huang H Lopez R Magrane M Martin MJ Natale DA O'Donovan C Redaschi N Yeh LSL UniProt: the Universal Protein knowledgebase. Nucleic Acids Res 2004 32 115 119 10.1093/nar/gkh131 14681372 PMC308865

[B59] *Antonospora locustae *Genome Database http://gmod.mbl.edu/perl/site/antonospora01

[B60] Edgar RC MUSCLE: multiple sequence alignment with high accuracy and high throughput. Nucleic Acids Res 2004 32 1792 1797 10.1093/nar/gkh340 15034147 PMC390337

[B61] Talavera G Castresana J Improvement of phylogenies after removing divergent and ambiguously aligned blocks from protein sequence alignments. Syst Biol 2007 56 564 577 10.1080/10635150701472164 17654362

[B62] Ronquist F Huelsenbeck JP MrBayes 3: Bayesian phylogenetic inference under mixed models. Bioinformatics 2003 19 1572 1574 10.1093/bioinformatics/btg180 12912839

[B63] Tamura K Dudley J Nei M Kumar S MEGA4: Molecular Evolutionary Genetics Analysis (MEGA) software version 4.0. Mol Biol Evol 2007 24 1596 1599 10.1093/molbev/msm092 17488738

[B64] Kohany O Gentles AJ Hankus L Jurka J Annotation, submission and screening of repetitive elements in Repbase: RepbaseSubmitter and Censor. BMC bioinformatics 2006 7 474 10.1186/1471-2105-7-474 17064419 PMC1634758

[B65] Benson G Tandem repeats finder: a program to analyze DNA sequences. Nucleic Acids Res 1999 27 573 580 10.1093/nar/27.2.573 9862982 PMC148217

