# Supplementary material for: Draft genome sequence of the Daphnia pathogen Octosporea bayeri: insights into the gene content of a large microsporidian genome and a model for host-parasite interactions
Source: Genome Biol. 2009 Oct 6;10(10):R106. doi: 10.1186/gb-2009-10-10-r106 (PMC2784321; doi:10.1186/gb-2009-10-10-r106)
Supplement: Additional data file 2 — The 80 O. bayeri proteins with assigned functions and motifs that are absent in Enc. cuniculi. [file gb-2009-10-10-r106-S2.DOC]

**Additional data file 2:** *O.bayeri* proteins with assigned functions and motifs that are absent in *Enc. cuniculi*

| **Process/Function** | **Protein assigned** | **Contig** | ***E*-value^1^** | **Motifs^2^** |
| --- | --- | --- | --- | --- |
| **Cell growth, cell division and DNA synthesis** | | | | |
| Cell cycle | Cyclin-dependent kinase regulatory subunit CKS1 | 10051 | 3.00E-19 | CKS, Plasmod_MYXSPDY |
| Cell cycle control | Endoribonuclease Dicer | 10131 | 1.00E-26 | dsrm, DUF1821 |
| DNA repair and  recombination | DNA mismatch repair protein MSH5 | 933 | 3.00E-18 | MutS domains III & V, Protein phos-phatase 2C |
| DNA polymerase | DinB translesion DNA repair polymerase | 28683 | 8.00E-16 | impB/mucB/samB family, CGGC domain |
| Non-homologous  end-joining | Similar to DNA cross-link repair 1A | 3552 | 2.00E-26 | DNA repair metallo-beta-lactamase, SecD/SecF GG Motif |
| Other | Deoxyribodipyrimidine photo-lyase  Putative transposase (*Nosema bombycis*)  Putative reverse transcriptase  Putative endonuclease-reverse transcriptase  Similar to reverse transcriptase homolog (*Rhizopus oryzae*)  Putative transposase  Putative transposase  Putative transposase  Putative transposase | 2153  2073  9400  7773  22  1621  2334  10692  13032 | 2.00E-70  3.00E-55  4.00E-26  7.00E-16  8.00E-26  2.00E-19  7.00E-74  2.00E-53  9.00E-16 | FAD_binding_7, DNA_photolyase, Hairy_orange, ALAD  RVT_1, RVT_thumb, UVR, RnaseH  RVT_1  RVT_1  RVT_1  N.A.  RVT_1  RVT_1  RVT_1 |
| **Cell rescue, defense, cell death and aging** | | | | |
| Detoxification | Glutathione reductase (NADPH) | 9316 | 1.00E-13 | Pyr_redox, Pyr_redox_2 |
| **Cellular organization and biogenesis** | | | | |
| Cell surface | Hypothetical spore wall protein (*Nosema bombycis*) | 860 | 3.00E-16 | DnaJ |
| Actin binding  (calponin domain) | Similar to AGR069Cp (*Ashbya gossypii*) | 14731 | 4.00E-17 | CH , TPR_MLP1_2, Ax_dynein_light |
| **Energy** | | | | |
| Cellular respiration | Alternative oxidase | 18332 | 4.00E-27 | AOX |
| Trehalose metabolism | Trehalose synthase | 12812 | 5.00E-60 | Glycos_transf_1 |
| **Intracellular transport** | | | | |
| Sterol Carrier activity | Oxysterol-binding protein | 996 | 4.00E-19 | PH domain, Oxysterol_BP |
| Magnesium carrier | CorA-like Mg2+ transporter protein | 4096 | 2.00E-70 | CorA |
| Sterol carrier activity | Similar to oxysterol-binding protein (*Schizosaccharomyces japonicus*) | 1782 | 5.00E-14 | Oxysterol_BP |
| **Metabolism** | | | | |
| Nitrogen and Sulfur | L-asparaginase | 7828 | 2.00E-16 | Asparaginase |
| Fatty acids | Acetyl-CoA carboxylase | 13002 | 6.00E-91 | Carboxyl_trans (x3) |
| Sphingolipids | Ceramide cholinephosphotransferase  Dihydroderamide delta-4 desaturase | 16842  587 | 7.00E-15  1.00E-51 | ATP-synt_A, PAP2  FA_desaturase |
| Glycerolipids | Diacylglycerol acyltransferase | 2535 | 1.00E-63 | LACT |
| Glycerophospholipids | Phosphatidylserine synthase 2  LYPLA2; lysophospholipase II  Phosphatidylserine decarboxylase | 5573  5529  18566 | 3.00E-53  4.00E-17  6.00E-49 | PSS  Abhydrolase_2  PS_Dcarbxylase |
| Other lipids | Elongation of very long chain fatty acids protein 3 | 11225 | 5.00E-59 | ELO |
| Purine | Nucleoside-diphosphatase  AMP deaminase  Adenosine kinase | 7640  20277, 15557  6058 | 1.00E-12  2.00E-81  1.00E-21 | GDA1_CD39  A_deaminase  AK, pfkB family carbohydrate kinase |
| Pyrimidine | dCMP deaminase | 307 | 8.00E-38 | dCMP_cyt_deam_1 |
| Glutathione | Glutamate-cysteine ligase catalytic subunit  Glutathione synthase | 9219  11491 | 2.00E-25  1.00E-18 | GCS  GSH_synth_ATP |
| Biotin | Biotin-[acetyl-CoA-carboxylase] ligase | 8941 | 2.00E-18 | BPL_LipA_LipB |
| Retinol | Retinol dehydrogenase 11 | 2396 | 2.00E-36 | Adh_short |
| Pantothenate and CoA | Pantothenate kinase | 24282 | 2.00E-13 | ROK, Funmble |
| Secondary metabolites | Carboxylesterase | 11789 | 4.00E-16 | Abhydrolase_2 |
| **Protein destination** | | | | |
| N-Glycan biosynthesis | UDP-N-acetylglucosamine-dolichyl-phosphate N-acetylglucosaminephosphotransferase  Beta-1,4-mannosyltransferase  Alpha-1,3/alpha-1,6-mannosyltransferase  ALG13; beta-1,4-N-acetylglucosaminyltransferase | 11862  7349  4055, 3192  6773 | 1.00E-48  6.00E-14  4.00E-22  1.00E-13 | Glycos_transf_4  GT1_ALG1  Glycos_transf_1  Glyco_tran_28_C |
| Glycosylphosphatidylinositol(GPI)-anchor biosynthesis | Phosphatidylinositol glycan, class B | 7186 | 3.00E-19 | Glyco_transf_22, |
| oligosaccharyl transferase activity | Oligosaccharyl transferase STT3 subunit | 11266 | 1.00E-32 | STT3 |
| protein amino acid farnesylation | Protein farnesyltransferase subunit beta  Prenyltransferase alpha subunit | 2251  6065 | 2.00E-47  3.00E-11 | Prenyltrans  PPTA |
| Ubiquitin carboxy-terminal hydrolase | Ubiquitin C-terminal hydrolase (*Ent. bieneusi*)  Ubiquitin carboxyl-terminal hydrolase (*Babesia bovis*)  Similar to ubiquitin C-terminal hydrolase Ubp8 | 691  3300  20999 | 7.00E-04  5.00E-05  4.00E-11 | Peptidase C19R  Peptidase C19R  Peptidase C19R |
| TIP60 complex (histone acetyl-transferase) | Similar to hypothetical protein EBI_25556 | 32199 | 8.00E-27 | MRG |
| **Protein synthesis** | | | | |
| Ribosomal protein | Large subunit ribosomal protein L14e | 647 | 2.00E-12 | Ribosomal_L14e |
| Other translation proteins | Eukaryotic translation initiation factor 2C | 25265 | 5.00E-21 | Piwi domain |
| **Transcription** | | | | |
| RNA polymerase | DNA-directed RNA polymerase I sub. A2  RNA dependent RNA polymerase | 23062  27820 | 1.00E-12  9.00E-12 | RNA_pol_Rpb2_3  RdRP |
| rRNA processing | DEAD box helicase | 8254 | N.A. | DEAD |
| RNA splicing factor | Similar to *Saccharomyces cerevisiae* CLF1 | 8110 | 3.00E-22 | HAT |
| RNA binding | Putative RNA binding protein | 18811 | N.A. | RRM_1 |
| Repressor of transcription | Negative regulator of differentiation 1 (Nrd1) | 8306 | 5.00E-31 | Ribonuc_P_40, RRM_1, ArsR |
| Transcription initiation | Similar to hypothetical protein An15g03490 (*Aspergillus niger*)  Transcription initiation protein SPT3 | 19691  16301 | 5.00E-04  2.00E-29 | HLH  TFIID-18kDa |
| RNA Capping | TrimethylGuanosine synthase  TrimethylGuanosine synthase (paralog) | 9671  1592 | 5.00E-13  4.00E-21 | Methyltransf_15  Methyltransf_15 |
| tRNA transcription | tRNA guanosine-2'-O-methyltransferase TRM11  tRNA (adenine-N1-)-methyltransferase | 17272  16590 | 5.00E-20  4.00E-12 | UPF0020  GCD14 |
| Histone methyltranferase | Similar to trithorax protein ash2 | 5688 | 1.00E-31 | SPRY domain |
| RNA processing and modification | Similar to YLR117Cp-like protein (*S. cerevisiae*) | 8058 | 4.00E-13 | HAT |
| **Transport facilitation** | | | | |
| Ion transporters | Ca2^+^-transporting ATPase  Cation-transporting ATPase  Cu2^+^-exporting ATPase | 521  15347  888 | 5.00E-122  N.A.  2.00E-16 | Cation ATPase N&C E1-E2 ATPase, Hydrolase  E1-E2 ATPase  E1-E2 ATPase, Hydrolase |
| **Unknown or involved in multiple processes** | | | | |
| Unclassified | aarF domain-containing kinase  Similar to hypothetical protein FG04269.1 (*Gibberella zeae*)  Nuclease (*S. cerevisiae*)  Leucine-rich repeats (LRRs), ribonuclease inhibitor (RI)-like subfamily  MET-10+related protein-like (ISS) [*Ostreococcus tauri*]  Methyltransferase-like protein 2  Putative hydrolase-like protein [*Antonospora locustae*]  Similar to hypothetical protein CC1G_07102 [*Coprinopsis cinerea* okayama) | 21838  8628  3807  10271  4094  6342  7662  10683 | 5.00E-19  4.00E-13  1.00E-26  2.00E-13  4.00E-19  2.00E-20  2.00E-10  1.00E-11 | ABC1  FH2  Snase  LRR_1  Met_10  Methyltransf_11  N.A.  N.A. |

^1^ BlastX homology searches against the NCBI non-redundant database. ^2^ According to Pfam or NCBI Conserved Domain Database searches. N.A.: not available in the current gene databases.
