# Supplementary material for: Draft genome sequence of the Daphnia pathogen Octosporea bayeri: insights into the gene content of a large microsporidian genome and a model for host-parasite interactions
Source: Genome Biol. 2009 Oct 6;10(10):R106. doi: 10.1186/gb-2009-10-10-r106 (PMC2784321; doi:10.1186/gb-2009-10-10-r106)
Supplement: Additional data file 4 — Sequences of the six introns identified in O. bayeri. [file gb-2009-10-10-r106-S4.DOC]

**Additional data file 4:** List and sequence of the 6 introns identified in *Octosporea bayeri* and homologous to *E.cuniculi*

L19 :5’-GTAAGTAAATTTATTCTCTTTACTAATTTTTAG-3’

L27a : 5’-GTAAGTAAATATTTGTATTAATTTTTAG-3’

L37a: 5‘- GTAAGTTTTACAATTTAAAAATAATTTTAG-3’

L37 : 5’- GTAAGTCATATAATTTTTAATTTTAG-3‘

L39 : 5’- GTAAGTGATTTTATTTAATTTTAG-3‘

s26 : 5’- GTAAGTTTTTAATACATTTTTCTTATTTAAG-3‘
