# Supplementary figures and images for: Draft genome sequence of the Daphnia pathogen Octosporea bayeri: insights into the gene content of a large microsporidian genome and a model for host-parasite interactions
Source: Genome Biol. 2009 Oct 6;10(10):R106. doi: 10.1186/gb-2009-10-10-r106 (PMC2784321; doi:10.1186/gb-2009-10-10-r106)

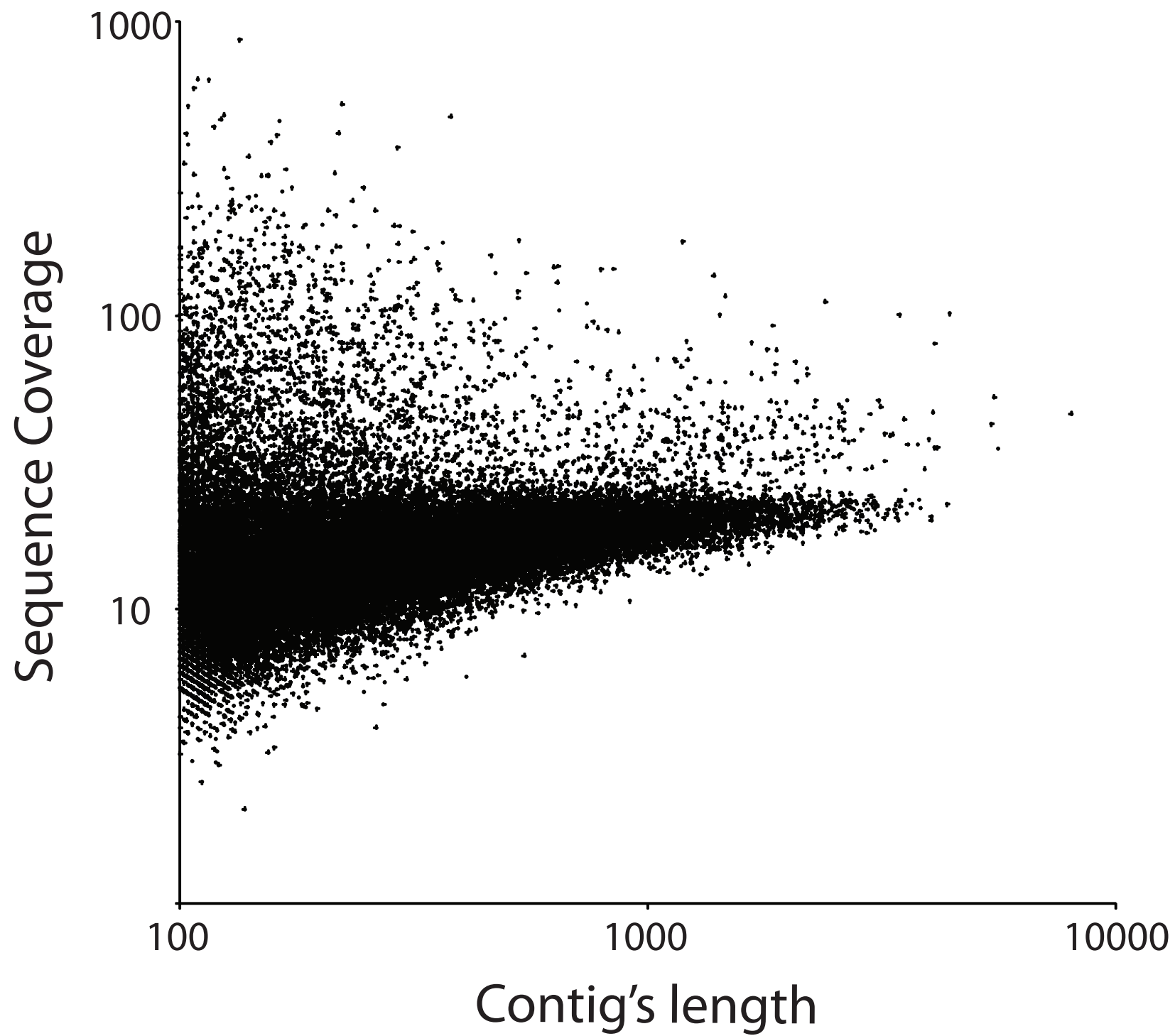

Supplement: Additional data file 6 — The number of contigs used in this study and their respective sequence coverage. [file gb-2009-10-10-r106-S6.PDF]
